# Host–pathogen interactions upon *Candida auris* infection: fungal behaviour and immune response in *Galleria mellonella*

**DOI:** 10.1080/22221751.2021.2017756

**Published:** 2021-12-21

**Authors:** Victor Garcia-Bustos, Javier Pemán, Alba Ruiz-Gaitán, Marta Dafne Cabañero-Navalon, Ana Cabanilles-Boronat, María Fernández-Calduch, Lucía Marcilla-Barreda, Ignacio A. Sigona-Giangreco, Miguel Salavert, María Ángeles Tormo-Mas, Amparo Ruiz-Saurí

**Affiliations:** a Department of Internal Medicine and Infectious Diseases, University and Polytechnic La Fe Hospital, Valencia, Spain; b Severe Infection Research Group, Health Research Institute La Fe, Valencia, Spain; c Department of Pathology, Faculty of Medicine and Dentistry, University of Valencia, Valencia, Spain; d Department of Medical Microbiology, University and Polytechnic La Fe Hospital, Valencia, Spain

**Keywords:** *Candida auris*, pathogenicity, host–pathogen interactions, virulence, filamentation, *Galleria mellonella*, immunopathogenesis

## Abstract

*Candida auris* has globally emerged as a multidrug-resistant fungus linked to healthcare-associated outbreaks. There is still limited evidence on its virulence, pathogenicity determinants, and complex host–pathogen interactions. This study analyzes the *in vivo* fungal behaviour, immune response, and host–pathogen interactions upon *C. auris* infection compared to *C. albicans* and *C. parapsilosis* in *G. mellonella.* This was performed by immunolabelling fungal structures and larval plasmatocytes and using a quantitative approach incorporating bioinformatic morphometric techniques into the study of microbial pathogenesis. *C. auris* presents a remarkably higher immunogenic activity than expected at its moderate degree of tissue invasion. It induces a greater inflammatory response than *C. albicans* and *C. parapsilosis* at the expense of plasmatocyte nodule formation, especially in non-aggregative strains. It specifically invades the larval respiratory system, in a pattern not previously observed in other *Candida* species, and presents inter-phenotypic tissue tropism differences. *C. auris* filaments *in vivo* less frequently than *C. albicans* or *C. parapsilosis* mostly through pseudohyphal growth. Filamentation might not be a major pathogenic determinant in *C. auris,* as less virulent aggregative phenotypes form pseudohyphae to a greater extent. *C. auris* has important both interspecific and intraspecific virulence and phenotype heterogeneity, with aggregative phenotypes of *C. auris* sharing characteristics with low pathogenic species such as *C. parapsilosis.* Our work suggests that *C. auris* owns an important morphogenetic plasticity that distinguishes it from other yeasts of the genus. Routine phenotypic identification of aggregative or non-aggregative phenotypes should be performed in the clinical setting as it may impact patient management.

## Introduction

*Candida auris* is an emergent fungal pathogen which, because of its multi-drug resistance [1–3], difficult identification by conventional microbiology techniques [4], high transmissibility, and environmental adaptability [5,6], has been associated with large outbreaks of healthcare-related invasive infections with a high mortality rate worldwide [2,5,7–9]. Consequently, in 2019, the Centers for Disease Control (CDC) considered it to pose an urgent threat to public health in the context of multi-drug resistant organisms [10,11].

To date, there is still limited evidence on *C. auris* virulence, pathogenicity determinants, and the complex host–pathogen interactions. Pathogenicity has been recently studied in several animal models such as *Galleria mellonella* [12–18], *Caenorhabditis elegans* [18], and mice [15]. However, it appears to be highly diverse, with some authors describing a comparable or even higher degree of pathogenicity to that of *C. auris* [12,13] contrarily to other reports [14,15]. High interspecific but also intra-species virulence heterogeneity seems to be an inherent characteristic of *C. auris* infection, as it also differs between aggregative and non-aggregative phenotypes, strains, and clades [16,19,20].

Despite the first insights into its cellular, molecular, and genetic pathogenicity determinants have been provided [13,20–22], histological data on *in vivo* host–pathogen interactions are also scarce. Some works in both murine and invertebrate animal models have superficially described the tissue fungal burden after infection [15,16,18,19,22–24]. Nevertheless, histological studies afford little evidence on fungal invasiveness, distribution, burden, and morphology, as well as host immune and cell biological response upon *C. auris* infection. Despite plasmatocytes are the responsible cells for fungal phagocytosis [25], different hemocyte subpopulations have not been analyzed nor quantified. Furthermore, no quantitative data have been reported to date beyond subjective, purely qualitative depictions using a very limited number of strains, individuals, and samples. Morphometry has been widely used in several fields of medical research such as cancer or ischemic heart disease to provide valuable quantitative histological information [26,27]. Indeed, immunofluorescence and immunohistochemistry have improved these techniques, enabling the differentiation of specific cell types.

To tackle this problem, we incorporated for the first time in the study of fungal pathogenesis the body of knowledge of morphometrics to quantitatively analyze the *in vivo C. auris* specific traits and mechanisms of invasion as well host cell and immune response by immunolabelling fungal structures and larval plasmatocytes in a *G. mellonella* infection model, compared to *Candida albicans* and *Candida parapsilosis* as high and low pathogenicity controls, respectively.

## Material and methods

### Selection and processing of fungal strains

Two strains of *C. auris* (CJ175 and CJ101), 1 strain of *C. albicans* (reference strain ATCC SC5314), and 1 strain of *C. parapsilosis* (reference strain ATCC 22019) were randomly selected and used for the essays. *C. auris* strains were isolated from clinical samples obtained from blood cultures of patients admitted to surgical or medical intensive care units (ICU) of the University and Polytechnic La Fe Hospital (Valencia, Spain) who developed candidemia, and kept at −80°C until use.

Blood cultures were processed with a BacT/ALERT^TM^ VIRTUO^TM^ automated microbial detection system. Definitive identification of *C. auris* was performed by internal transcribed spacer (ITS) sequencing using ITS4-ITS5 primers following the protocol used by Bellemain et al. [28] The sequencing technique was performed using GenomeLabTM GeXP kits (Beckman Coulter, Fullerton, CA, USA) and the sequences obtained were compared with those of Microbial Genomes BLAST (http://www.ncbi.nlm.nih.gov/guide/sequence-analysis/) and Mycobank Database. (*C. auris* CJ175 GenBank accession number: KJ126759.1, 96% similarity with sequence no AB375772. *C. auris* CJ101 accession number KC692045.1, 95% similarity with sequence no AB375772). For DNA extraction, inoculum was prepared from a 48-h culture grown on Sabouraud agar with chloramphenicol (Oxid Madrid, Spain) by resuspending 2–3 colonies in 800 µL of lysis buffer (Tris HCl 0.06M, EDTA 0.1M, NACl 1M, BrijTM −58 0.5%, sodium deoxycholate 0.2%, Lauryl Sarcosyl 0.5%). Once homogenized, 80 µL of Proteinase K (Qiagen, France) was added and incubated for 45 min at 56°C. Three freeze/thaw cycles were performed at −80°C/95°C for 1 min. Finally, extraction was performed using the DNeasy Blood and Tissue kit (Qiagen) on the QIAsymphony® automated extraction system (Qiagen, France). All isolates were sent for confirmation and complete genome sequencing to the Mycology Reference Laboratory of the National Microbiology Centre of the Carlos III Health Institute (ISCIII). Aggregative or non-aggregative phenotypes were determined by documenting immediate yeast aggregation at 200× magnification after vortexing for 3-minutes 1 mL of sterile saline with a concentration of 10^8^ CFU/mL.

### Infection model in *G. mellonella*

Healthy sixth instar larvae of *G. mellonella* weighing 250–350 mg (TruLarv™, BioSystems Technology Ltd., UK) were selected. Larvae were decontaminated upon arrival using 70% ethanol and were kept in groups of 10 in Petri dishes at 15°C and dark conditions until inoculation.

Fungal isolates were grown on Sabouraud’s agar for 24 h at 37°C. Individual colonies were collected with sterile plastic loops, washed twice in sterile phosphate-buffered saline (PBS), counted with a TC20^TM^ Automated Cell Counter (BioRad Laboratories, France), and adjusted to 10^5^ CFU/μL in sterile PBS. A standard inoculum of 10 μL of the solution (10^6^ CFU) was chosen [12,16].

Thirty larvae were inoculated with 10^6^ CFU of *C. albicans* in the left rear proleg using a 10 μL Hamilton syringe with a 26-gauge blunt needle. Another group of 30 larvae was infected with 10^6^ CFU of *C. parapsilosis*. Eighteen larvae were inoculated with 10^6^ CFU of the *C. auris* strain CJ101, and 15 larvae with 10^6^ CFU of the *C. auris* strain CJ175 by intrahemocelic injection of 10 μL of the solution (10^6^ CFU) Groups of 10 infected larvae were placed in Petri dishes and were incubated at 37°C. Further analyses were blinded by tagging every Petri dish with an individual code. Larvae were euthanized after 24 h of incubation following the AVMA Guidelines for the euthanasia of animals. They were firstly anesthetized through immersion in 5% ethanol and later euthanized by immersion in a solution of neutral-buffered 10% formalin. To preserve tissue architecture while allowing formalin cuticular penetration and tissue diffusion, larvae were fixed for 28 days as previously described [16].

### Tissue processing and immunohistochemical staining

Each larva was midsagittally sectioned and embedded in paraffin. Histological 3–4 μm thick sections were obtained with the microtome. Immunohistochemistry (IHC) with specific antibodies against *Candida* spp. and *G. mellonella* plasmatocytes was performed.

#### *Candida* spp. immunostaining

Midsagittal tissue sections were deparaffinized and rehydrated through graded alcohols. All samples were tagged for identification with the number of the larva and Petri dish blinded coding system. The primary murine polyclonal IgG anti-*C. albicans* antibody (PA1-27158, Thermo Fisher Scientific, Waltham, MA, USA) was chosen to specifically label fungal structures, as it was shown to have cross-reactivity with *C. auris* and *C. parapsilosis* strains. Tissue sections were incubated with the primary antibody at a dilution of 1:20,000 at room temperature for 45 min. Amplification of the primary antibody signal was carried out by 45 with polyclonal goat anti-rabbit immunoglobulins/HPR as secondary antibody for 45 min (Thermo Fisher Scientific, Waltham, MA). Tissues were then counterstained with hematoxylin–eosin.

#### *G. mellonella* novel plasmatocyte immunostaining

We specifically immunolabeled *G. mellonella* plasmatocytes. Following deparaffination and rehydration, a murine monoclonal IgG2b MS13 antibody against *Manduca sexta* plasmatocyte beta-integrin (Developmental Studies Hybridoma Bank, The University of Iowa, Iowa City, IA, USA) was used for *G. mellonella* plasmatocyte immunostaining. It was shown to cross-react and adequately immunolabel plasmatocytes of *G. mellonella* larvae. The primary MS13 antibody was incubated at a dilution of 1:20 for 45 min at room temperature. Subsequently, sections were incubated for 45 min with the secondary polyclonal goat anti-rabbit immunoglobulins/HPR and counterstained with hematoxylin–eosin.

### Histopathological evaluation and morphometric analysis

All samples were assessed using the optic Leica DMD108 digital microimaging network instrument (Leica Microsystems, Wetzlar, Germany). Entire midsagittal larval sections were analyzed. The whole larval area was mapped by taking photomicrographs at 40× magnification for quantification of fungal density, 100× magnification for plasmatocyte analysis, and 200× up to 630× to evaluate *in vivo* fungal morphology, distribution, and degree of phagocytosis. The computerized morphometric study was carried out using the software Image Pro-Plus 7.0 (Media Cybernetics, Silver Spring, MD, USA).

For the study of the fungal load, tissue distribution, and *in vivo* cellular characteristics and host–pathogen interactions, several variables were analyzed:

- Fungal density: percentage resulting from the sum of the total area (μm^2^) occupied by yeast in all analyzed micrographs per larva, divided by the complete area of the larval midsagittal section (μm^2^).
- Fungal distribution: the main tissue distribution of the fungal pathogens -namely the digestive tract, fat tissue, hemolymph, tracheae, respiratory system, and uncharacterizable inflammatory tissue- was recorded.
- Fungal morphology: the presence of pseudohyphae or true hyphae was distinctively registered.
- Phagocytosis and presence of intracellular yeasts.

Further magnification captures up to 630× magnification were performed for the last two parameters, to better characterize fungal morphology and intracellular structures, whenever present.

The larval immune response was assessed in the whole larval section, based on the plasmatocyte-specific immunolabelling. The following morphometric parameters were studied:

- Plasmatocyte density: the number of plasmatocytes was recorded in each 100× magnification photomicrograph. In large immune aggregates, a watershed segmentation algorithm was applied to enable cellular quantification. The sum of the number of plasmatocytes in all micrographs of each larva was divided by the complete larval area. Results were expressed in plasmatocytes per mm^2^.
- Mean plasmatocyte diameter and area per 100× magnification micrograph.
- Plasmatocyte roundness: the roundness of each plasmatocyte has been associated with the degree of cellular activation and was determined by the following formula:
$$\frac{Plasmatocyte\;\;Perimeter^{2}}{4\times \pi \times Plasmatocyte\;Area}$$

Completely circular objects present a roundness = 1; other shapes present a roundness largely > 1 and would correspond to activated cells with an irregularly shaped cell membrane.

- Plasmatocyte distribution: the main tissue distribution of plasmatocytes -namely the digestive tract, fat tissue, hemolymph, tracheae, respiratory system, and uncharacterizable inflammatory tissue- was recorded per 100× magnification micrograph.
- Plasmatocyte nodulation: nodules were defined as the aggregation of 5 or more plasmatocytes. The presence of nodules was registered in each 100× magnification photomicrograph.

### Statistical analysis

The descriptive statistics, graph representations, and hypothesis contrasts were performed with the statistical software R version 4.0.0 (R Development Core Team, 2020). Continuous variables were expressed as mean and standard deviation (SD). Categorical variables were expressed as a percentage. The level considered to indicate statistical significance was *p* < 0.05. The descriptive statistics for each morphometric parameter were calculated and quantile-quantile (QQ) plots were performed to assess normality for each variable. The Levene test was used to assess the equality of variances in quantitative variables. In the comparison of frequencies of qualitative variables, Pearson’s *χ*^2^ test was used, and Bonferroni *p*-value correction was applied for multiple comparisons. In the case of quantitative variables following normal or non-normal distributions, ANOVA and Kruskal–Wallis tests were chosen, respectively.

## Results

The two *C. auris* isolates were obtained from critically ill patients with candidemia. CJ101 showed an aggregative phenotype while CJ175 was a non-aggregative strain.

### Morphometric analysis of the plasmatocyte response to *C. auris* infection in *G. mellonella*

#### *C. auris* induces a higher tissue density of plasmatocytes than *C. albicans* and *C. parapsilosis* at the expense of nodule formation

After analyzing more than 1,200 100× magnification micrographs and mapping all the tissue area in larval midsagittal sections, the tissue density of plasmatocytes after infection by *C. auris* was almost 4-fold higher than after infection by *C. albicans* and *C. parapsilosis* (*p* = 5.22 × 10^−9^), with a mean of approximately 24 plasmatocytes per mm^2^ (Table 1). No significant differences were observed between larvae infected with *C. albicans* and *C. parapsilosis* (*p* = 1). The presence of inflammatory hemocyte aggregates was significantly higher after infection by *C. auris* than after infection by *C. albicans* and *C. parapsilosis* (*p* = 8.32 × 10^−7^)*.* In 62.8% of the micrographs in the *C. auris* infected group plasmatocytes aggregated forming inflammatory nodules, while in the larvae infected by both *C. albicans* and *C. parapsilosis* they barely reached 50% (Table 1).

**Table 1.** Summary of the main morphometric parameters.

Parameter	Species			*p*-value
	*C. albicans*	*C. auris*	*C. parapsilosis*	
N_1_	30	33	30	NA
N_2_	307	639	311	NA
Density (cells/mm^2^) mean – SD	8.51–7.03	23.83–13.68	8.77–7.76	5.22 × 10^−9^
Diameter (μm) mean – SD	6.66–1.36	7.23–1.22	7.43–1.32	3.75 × 10^−14^
Area (μm^2^) mean – SD	41.44–18.79	49.62–18.09	52.01–19.28	6.08 × 10^−13^
Roundness mean – SD	2.15–0.83	1.95–0.50	1.87–0.45	1.3 × 10^−8^
Nodulation n – %	152–49.5	401–62.8	145–46.6	8.32 × 10^−7^

N_1_: number of larvae. N_2_: number of analyzed micrographs. NA: not applicable. SD: standard deviation.

Furthermore, the *C. auris* capability of inducing a larger hemocyte density was independent of the phenotype and in both cases was higher than 20 plasmatocytes per mm^2^ (26.22 – SD 14.41 and 21.83 – SD 1.11; *p* = 1). However, their tendency to form nodules was significantly lower in the aggregative strain than in the non-aggregative strain, with a nodulation frequency of 57.3% of the micrographs for aggregative *C. auris* versus 70.9% for non-aggregative *C. auris* (*p* = 0.004) (Figure 4C and D).

#### Plasmatocytes of larvae infected by *C. auris* and *C. parapsilosis* have a lower size and membrane irregularity than *C. albicans*

More than 34,500 plasmatocytes were analyzed. On the one hand, significant differences were observed in the mean plasmatocyte area and diameter between *C. albicans* and *C. auris,* as well as between *C. albicans* and *C. parapsilosis* (*p* = 6.08 × 10^−7^ and *p* = 3.75 × 10^−14^, respectively). Plasmatocytes were significantly smaller in larvae infected with *C. albicans* than in those with both *C. auris* and *C. parapsilosis*. However, no significant differences were observed when comparing their mean area and diameter between larvae infected with *C. auris* and *C. parapsilosis* after Bonferroni *p*-value adjustment for multiple comparisons (*p* = 0.061).

**Figure 1.**
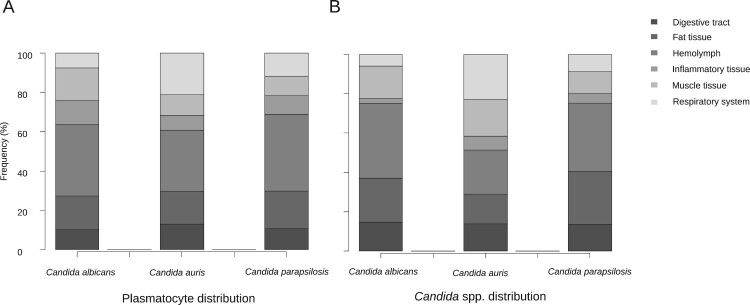
Differences of plasmatocyte and fungal tissue distribution after infection with *C. albicans*, *C. auris* and *C. parapsilosis*.

On the other hand, plasmatocyte roundness or membrane irregularity was significantly lower after *C. albicans* infection than after both *C. auris* and *C. parapsilosis* infection (*p* = 2.969 × 10^−6^ and 3.3 × 10^−8^, respectively) while it did not differ between the last two species (*p* = 0.24) (Figure 1A). The mean roundness values and SD observed for each group are presented in Table 1. No differences were observed when comparing aggregative and non-aggregative phenotypes (*p* = 1).

#### Tissue tropism of *G. mellonella* plasmatocytes in response to infection varies depending on the species of *Candida*

**Figure 2.**
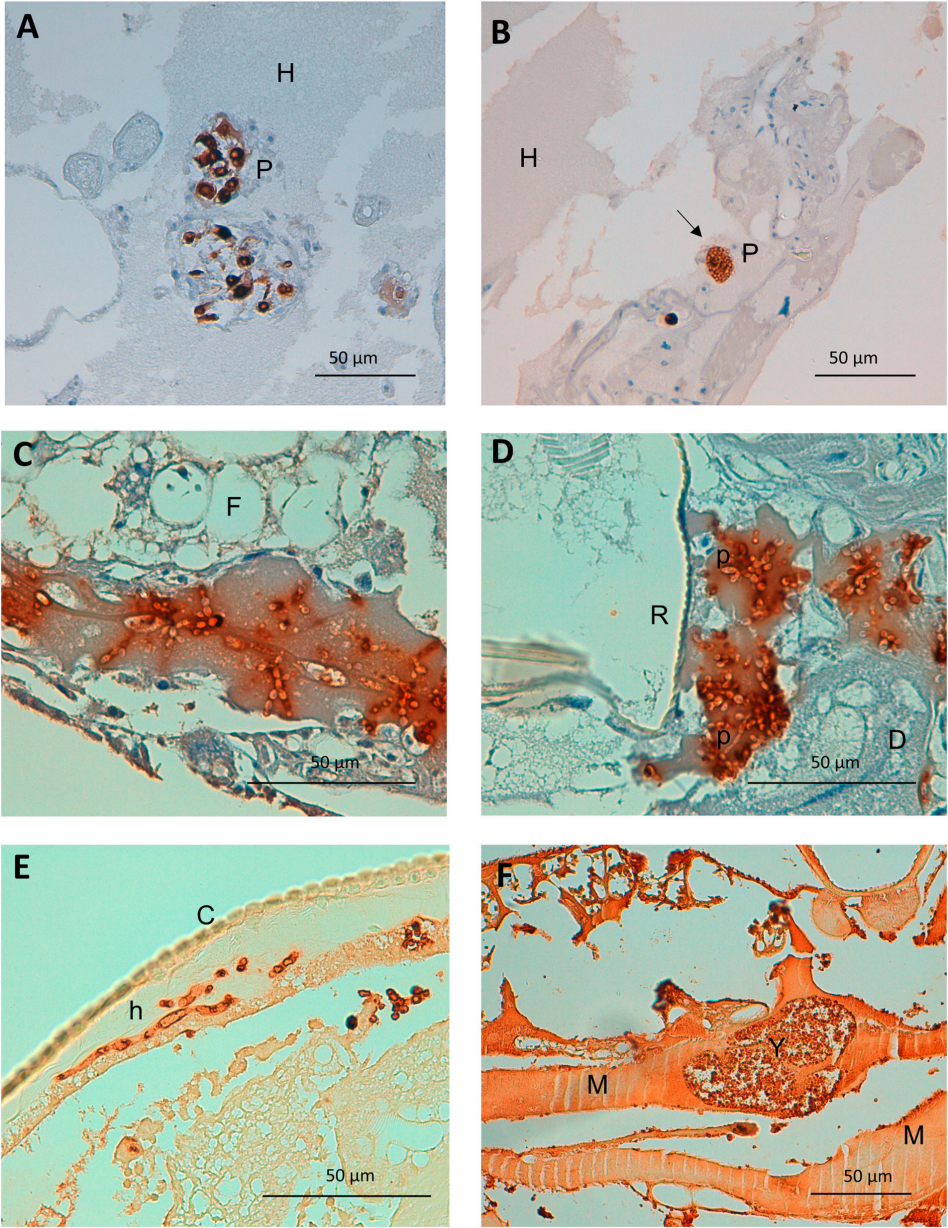
Detail on fungal invasiveness and plasmatocyte characteristics as a response to *Candida* spp. infection in *G. mellonella*. (A) Detail on small nodule of activated irregular plasmatocytes. Anti-MS13 antibody. CJ101 strain. 400× magnification. (B) Large quiescent plasmatocyte containing many phagocyted yeasts. Anti-MS13 antibody. CJ175 strain. 400× magnification. (C) *C. auris* pseudohyphae in adipose tissue and hemolymph. Anti-*C. albicans* antibody. CJ101 strain. 630× magnification. (D) *C. auris* pseudohyphal forms with a peri-respiratory distribution. Anti-*C. albicans* antibody. CJ101 strain. 630× magnification. (E) *C. albicans* true hyphae located subcuticularly, Anti-*C. albicans* antibody. SC5314 strain. 630× magnification. (F) Large yeast aggregate invading muscle tissue. Anti-*C. albicans* antibody. SC5314 strain. 400× magnification. C, cuticle; D, digestive tissue; F, fat tissue; H, hemolymph; M, muscle tissue; P, plasmatocyte; p, pseudohyphae; R, respiratory tissue; Y, yeasts.

**Figure 3.**
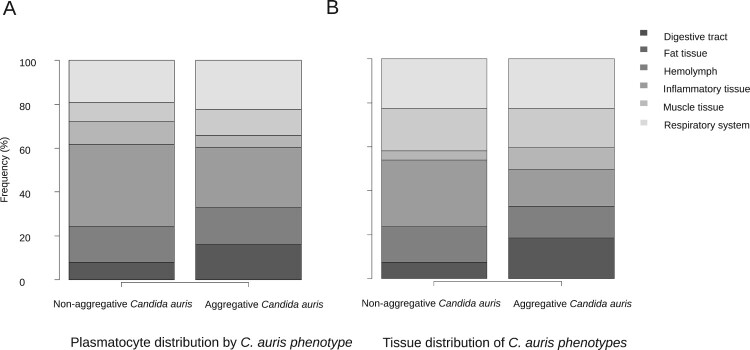
Differences of plasmatocyte and fungal tissue distribution after infection with non-aggregative and aggregative *C. auris*.

Hemolymphatic plasmatocyte distribution was predominant in all three *Candida* species (Figure 1A). More than 30% of photomicrographs mainly showed plasmatocytes within the hemolymph. A significant differential distribution was observed, however, when analyzing other larval tissues. Whilst in larva infected with *C. albicans* and *C. parapsilosis* the fat tissue distribution was the second most frequently observed distribution (17.3% and 19.3%, respectively), plasmatocytes of larva einfected with *C. auris* infected were seen in close relation with the respiratory system in more than 20% of the photomicrographs (Figure 1A). *C. albicans* showed remarkable muscle tissue inflammation (16.6%) (Figures 1A and 2F). These differences were statistically significant (*p* = 5.602 × 10^−7^). Both non-aggregative and aggregative *C. auris* phenotypes showed similar tissue distribution patterns, with a discrete predominance of peri-intestinal plasmatocytes for aggregative *C. auris* (15.9% in aggregative *C. auris* vs. 8% in non-aggregative *C. auris*) and fewer hemolymphatic plasmatocyte observations (27.2% in aggregative *C. auris* vs. 37.5% in non-aggregative *C. auris*), as seen in Figure 3. The plasmatocyte distribution was partially superimposable to that observed for fungal structures, as seen in Figure 1, Figure 3, and Table 2.

**Table 2.** Plasmatocyte and *Candida* spp. in vivo tissue distribution.

Species	N_1_	N_2_	Distribution *n* (%)					
			Digestive tract	Fat tissue	Hemolymph	Inflammatory tissue	Muscle tissue	Respiratory system
Plasmatocyte tissue distribution								
* C. albicans*	30	307	31 (10.1)	53 (17.3)	111 (36.2)	38 (12.4)	51 (16.6)	23 (7.5)
* C. auris*	33	639	82 (12.8)	107 (16.7)	199 (31.1)	48 (7.5)	68 (10.6)	135 (21.1)
* C. parapsilosis*	30	311	33 (10.6)	60 (19.3)	121 (38.9)	30 (9.6)	30 (9.6)	37 (11.9)
*Candida* spp. tissue distribution								
* C. albicans*	30	741	109 (14.7)	165 (22.3)	282 (38.1)	18 (2.4)	123 (16.6)	44 (5.9)
* C. auris*	33	1267	176 (13.9)	190 (15)	287 (22.7)	88 (6.9)	236 (18.6)	290 (22.9)
* C. parapsilosis*	30	569	77 (13.5)	153 (26.9)	198 (34.8)	28 (4.9)	64 (11.2)	49(8.6)

N_1_: number of larvae. N_2_: number of analyzed micrographs.

### Analysis of fungal burden, distribution, and morphology after *C. auris* infection in *G. mellonella*

#### *C. auris* seems to produce an intermediate fungal burden between *C. albicans* and *C. parapsilosis*

A statistically non-significant trend was observed in the mean fungal density (*p* = 0.058). The mean percentage of the larval midsagittal section area infected was 1.26% (SD 3.00) for *C. albicans*, 0.51% (SD 0.62) for *C. auris,* and 0.19 (SD 0.15) for *C. parapsilosis.* However, this higher pathogenicity was mainly observed for non-aggregative *C. auris* phenotypes. The fungal density of the aggregative strain CJ101 did not differ from that observed for *C. parapsilosis*. The mean fungal density for the non-aggregative strain was 0.83% of the larval tissue area (SD 0.81) while for the aggregative strain was 0.25% (SD 0.18).

#### *C. auris* specifically invades *G. mellonella* respiratory tissue, with non-aggregative phenotypes mainly located in the hemolymph and aggregative phenotypes showing a more homogeneous distribution of intestinal predominance

**Figure 4.**
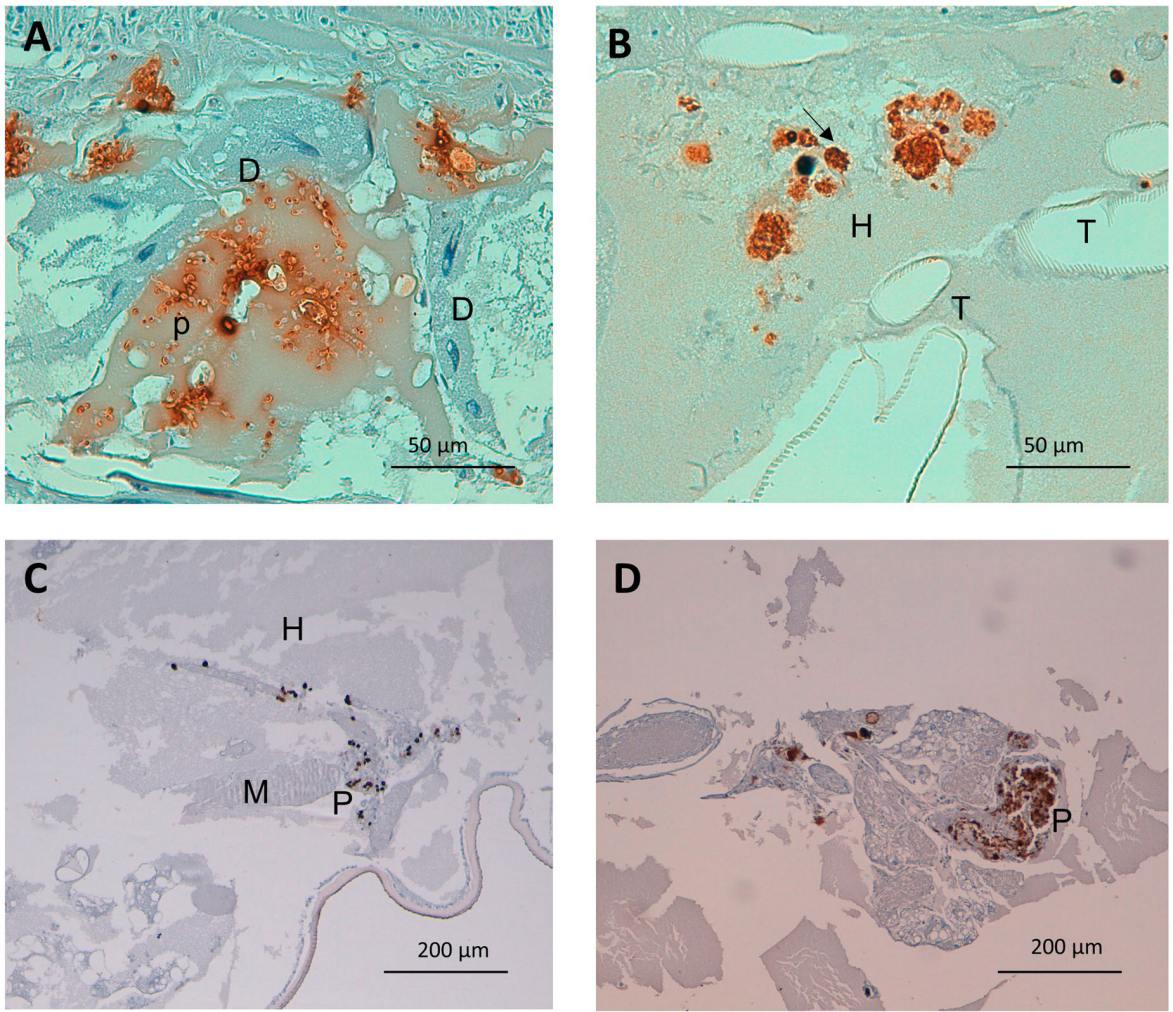
Detail on fungal and plasmatocyte characteristics as a response to *G. mellonella* infection by aggregative and non-aggregative phenotypes of *C. auris*. (A) *C. auris p*seudohyphae invading larval digestive epithelium, after infection with the aggregative strain CJ101. Anti-*C. albicans* antibody. 400× magnification. (B) Larval hemolymph containing yeasts of *C. auris*. Note the presence of intracellular yeasts within infected and plasmatocytes (arrow) undergoing cell lysis. Non-aggregative strain CJ175. Anti-*C. albicans* antibody. 400× magnification. (C) and (D) Small and large plasmatocyte nodules after infection with aggregative (CJ101) and non-aggregative (CJ175) strains of *C. auris,* respectively. Anti-MS13 antibody. 100× magnification. D, digestive tissue; H, hemolymph; P, plasmatocyte; T, respiratory tracheal system.

Large significant differences were observed regarding the tissue tropism of *C. auris* compared to *C. albicans* and *C. parapsilosis* (*p* = 2.2 × 10^−6^). The tissue invasion of *C. albicans* and *C. parapsilosis* was homogeneous, with most of the fungal load distributed in the hemolymph, followed by the larval adipose tissue and the intestinal tract. However, *C. auris* substantially invaded the larval respiratory system (Figures 1B and 2D), as seen in Table 2. Significant differences were observed between *C. albicans* and *C. parapsilosis* after Bonferroni *p*-value adjustment (*p* = 0.0048). *C. albicans* showed a muscle distribution more frequently than *C. parapsilosis.* Moreover, important differences were observed in the behaviour of different phenotypes (*p* = 2.2 × 10^−13^), as depicted in Figure 3. Although both aggregative and non-aggregative *C. auris* strains affected tracheal structures in 22.6% and 22.7% of micrographs (Figures 2D and 4B), respectively, non-aggregative *C. auris* was predominantly distributed through the larval hemolymph (30.5% of 594 micrographs) (Figure 4B), followed by tracheae, muscle cells (19.2%), fat tissue (16%), and intestinal tract (7.6%). For the aggregative strain, digestive structures were predominantly affected after respiratory tissue (18.6% of 758 micrographs) (Figure 4A), followed by muscle tissue (17.7%), hemolymph (16.8%), and adipose tissue (14.2%) (Figure 3). The tissue distribution of both phenotypes significantly differed from *C. albicans* and *C. parapsilosis* (*p* < 10^−12^).

#### *C. auris* can filament in vivo to a lesser extent than both *C. albicans* and *C. parapsilosis* and mainly in the form of pseudohyphal structures

Of 482 micrographs, 259 (35%) showed some degree of filamentation in larvae infected by *C. albicans.* In larvae with *C. auris* and *C. parapsilosis* infection, 16.3% (206/1267) and 27.6% (157/569) of micrographs presented filamented fungal structures (Figure 2C and D). These differences were statistically significant (*p* = 3.88 × 10^−21^).

On the contrary, no significant differences were observed in the degree of filamentation between aggregative and non-aggregative *C. auris* phenotypes, despite observing filamented forms in 23.2% of micrographs in the aggregative group versus 10.6% in the non-aggregative group.

Yeast form structures were the most frequently found *in vivo* in all three species, as seen in Table 3. Nevertheless, highly significant differences were seen in the cellular morphology (*p* = 1.97 × 10^−64^). *C. albicans* usually presented both pseudohyphal and true hyphal forms (Figure 1E) (17.5% and 17.3% of the photomicrographs, respectively), followed by *C. parapsilosis,* which mainly only developed pseudohyphae (26.5%). Finally, *C. auris* presented a lower frequency of filamentation and a majority of pseudohyphal forms (16.1% of pseudohyphae vs. 0.6% of true hyphae) both in aggregative and non-aggregative phenotypes (Figures 2C, 2D and 4A).

**Table 3.** Fungal morphology characteristics.

Species	N_1_	N_2_	Presence of filamentation		
			Yeast form *n* (%)	Pseudohyphae *n* (%)	True hyphae *n* (%)
*C. albicans*	30	741	483 (65.2)	130 (17.5)	128 (17.3)
*C. auris*	33	1267	1056 (83.3)	204 (16.1)	7 (0.6)
*C. parapsilosis*	30	569	410 (72.1)	151 (26.5)	8 (1.4)

N_1_: number of larvae. N_2_: number of analyzed micrographs.

#### *C. auris* non-aggregative phenotypes and *C. albicans* show a lower frequency of intracellular forms than aggregative *C. auris* strains and *C. parapsilosis*

The frequency of micrographs showing intracellular and intrahemocyte fungal forms was significantly higher in *C. parapsilosis* (35.1% of 569 micrographs), followed by *C. auris* (27.5% of 1,267 micrographs) and finally *C. albicans* (19.3% of 741 micrographs) (*p* = 8.467 × 10^−10^). These results run in parallel to the mean plasmatocyte diameter and area and may express larger sizes for those hemocytes with intracellular fungal forms. Furthermore, these differences were observed at the expense of a lower number of intracellular cells in *C. auris* non-aggregative phenotypes. Furthermore, plasmatocytes after infection with the non-aggregative phenotype strain usually presented a disrupted appearance (Figure 4B). The frequency of intracellular forms in the aggregative strain was similar to that observed in the *C. parapsilosis* group (36.1% of 758 micrographs).

## Discussion

This study has shown that *C. auris* behaves differently from other species of *Candida* in the invertebrate host, but also presents a remarkable inter-phenotypic heterogeneity. In this work we have analyzed the *in vivo* fungal behaviour, host cell and immune response, and host–pathogen interactions upon *C. auris* infection in *G. mellonella* by specifically immunolabelling fungal structures and plasmatocytes, through a novel quantitative approach incorporating morphometric techniques into the study of microbial pathogenesis.

The main findings of this work can be summarized as follows: (i) *C. auris* owns a remarkably higher immunogenic activity than expected at its moderate degree of tissue invasion, inducing greater inflammatory response than *C. albicans* and *C. parapsilosis* at the expense of plasmatocyte nodule formation, especially in non-aggregative strains. (ii) *C. auris* specifically invades the larval respiratory system and presents inter-phenotypic tropism differences, with a more homogeneous distribution of intestinal predominance for aggregative strains resembling other *Candida* species. (iii) *C. auris* filaments *in vivo* less frequently than *C. albicans* or *C. parapsilosis* mostly through pseudohyphal growth. Filamentation might not be a major pathogenic determinant in *C. auris,* as known less virulent aggregative phenotypes tend to form pseudohyphae to a greater extent. (iv) Hemocyte immune response is superimposable to fungal tissue distribution. Plasmatocytes phagocyte less frequently non-aggregative *C. auris* phenotypes and *C. albicans*, for which they present a higher degree of activation and membrane irregularity than aggregative *C. auris* and *C. parapsilosis*.

*C. auris* morphogenetic plasticity and distinct expression of virulence factors may account for its remarkable inter and intraspecific heterogeneity [16,22,29,30]. Several genetic and molecular pathogenicity determinants have been defined for *C. auris*, such as expression of hemolysin, lipases, secreted aspartyl proteinases (SAPs), phospholipases, adhesins, integrins, or biofilm-related genes (*IFF4, CSA1, PGA26*, among others) [31]. However, morphological plasticity plays a critical role in fungal virulence [30], namely by biofilm formation and hyphal growth for *Candida* species [32]. In the unique case of *C.* auris, *in vivo* phenotypic yeast aggregation and yet uncertain filamentation ability are thought to partly explain this diversity. Despite the existence of discordant data on the species pathogenicity depending on strains and possibly clades, showing both higher [12,13] and lower virulence compared to *C. albicans* [14–17,19], evidence is congruent on aggregative strains being less virulent. However, it was not until recently that we began to have data on the *C. auris* ability to produce pseudohyphae. Previously considered unable to filament [12], recent studies have described phenotypic switching to pseudohyphal forms after genotoxic stress [33], temperature changes and passage through mammalian [22], invertebrate organisms [13,16,30], or even *in vitro* [30].

To date, there is scarce histopathological information on *C. auris* pathogenesis, and limited evidence is biased owing to works using only subjective approaches with a very limited number of strains, individuals, and samples. Moreover, there are no *in vivo* studies devoted to analyzing fungal pathogeny and host immune and cell biological responses upon *C. auris* infection. Computerized morphometric techniques provide a mathematical description of fungal and immune cell forms and densities, offering a more direct understanding of host–pathogen interactions and behaviour upon infection.

Through this approach, we have identified *C. auris* as highly immunogenic when compared to *C. albicans* and *C. parapsilosis*, while showing an intermediate fungal density. This finding contrasts with previous studies of hemocyte count in hemolymph extracts, in which an inverse relationship with infectious fungi and hemocyte density was described [34]. This may be explained due to elevated solid tissue tropism and aggregation into large nodules. As hereby demonstrated and in concordance with histological studies in murine models [19], these nodules run in parallel to the fungal load distribution, as hereby demonstrated. Previous reports on neutrophil and macrophage inflammatory infiltrate in mammal organs upon *C. auris* infection might imply a common pathway for the innate immune response induction in invertebrates or superior organisms including humans [19,23].

Moreover, the particular traits of the tissue distribution of *C. auris* described in this work are in line with known highly frequent septic metastatic complications in patients with *C. auris* infection [6]. We have quantitatively demonstrated its previously suggested specific respiratory tropism in *G. mellonella* [16], which significantly differed between phenotypes. Although both phenotypes predominantly invaded larval tracheae, aggregative strains similarly reproduced *C. albicans* and *C. parapsilosis* distributions. However, we did not observe such an intestinal majority of fungal elements in *C. albicans* as previously reported in a qualitative manner [34].

On this line, several findings of this work advocate the lower virulence seen in aggregative phenotypes both in invertebrate and vertebrate animal models [12–16,19]. Aggregating *C. auris* forms induced plasmatocyte nodules to a lesser extent, but these immune cells did not appear to be less active than those seen upon non-aggregating *C. auris* infection. However, the tissue fungal burden of the aggregative phenotype was lower than in the non-aggregative phenotype and did not differ from that observed in *C. parapsilosis*: a known lower pathogenicity species of the genus [16,35,36]. Additionally, the aggregative phenotype presents a greater frequency of intracellular forms which, concerning its lower virulence, might imply either increased immune evasion mechanisms [31], lack of phagocytosis, or enhanced yeast “dumping” as yeast release after intracellular phagocyte killing [37] for non-aggregating strains. However, another striking finding of this work is the notably higher frequency of filamented forms in the aggregative group, which has also been previously described [30]. Filamentous forms have traditionally been associated with increased invasiveness and virulence [32]. Nevertheless, these findings make us hypothesize whether pseudohyphal growth in *C. auris* is not one of the main pathogenic determinants upon infection, especially after hyphal-related proteins implied in pathogenesis such as candidalysin (ECE1) have not been detected in some works [15]. These data raise an important matter for clinical practice. Usually, phenotypic characterization is not routinely determined in hospital-based clinical microbiology laboratories, but we suggest its routine characterization and providing the information to clinicians, as knowing the aggregating phenotype might have implications in patient management and treatment beyond antifungal sensitivity testing.

Some limitations of the study deserve mention. Despite analyzing more than 4,500 different magnification micrographs and covering the whole tissue area, around 30 larvae per group were included and this sample size could ideally be increased. Likewise, the most important limitation is the use of only 2 strains of *C. auris*, especially in the highly diverse context of *C. auris*, as previously discussed. *G. mellonella* has been identified as an interesting model due to its complex innate immune system with functional and anatomical similarities to those found in more complex organisms [38], but results must be carefully interpreted, and extrapolation might be limited. Further analyses with a greater number of strains, even randomly selecting tissue areas for experimental feasibility, should be performed. Furthermore, these findings should be correlated with genomic analyses, RT–PCR analysis of virulence gene mRNA expression, further phenotypic changes, phospholipase activity detection, and biofilm formation ability, among others, to fully explain its wide morphogenetic plasticity.

In conclusion, we have documented differential traits in fungal invasiveness, distribution, burden, and morphological plasticity as well as host immune and cell biological response for *C. auris* aggregative and non-aggregative phenotypes compared to other species by incorporating the methodology of morphometrics. *C. auris* is a potent immune inductor at the expense of nodule formation, with specific respiratory tropism and filamentation capability through pseudohyphal growth. However, filamentation does not seem to be as decisive for its pathogenesis as previously assumed. *C. auris* owns important both interspecific and intraspecific virulence and phenotype heterogeneity with aggregative phenotypes of *C. auris* sharing characteristics with low pathogenic species such as *C. parapsilosis.* Hence, our work suggests that *C. auris* owns an important morphogenetic plasticity that distinguishes it from other yeasts of the genus. Routine phenotypic identification of aggregative or non-aggregative phenotypes should be performed in the clinical setting as it may impact patient management. Further studies are needed to completely untangle the molecular pathways underlying this plasticity that endow this emergent pathogen with an unusual complexity.
